# Do Junior Doctors feel confident using Emergency Detention Certificates?

**DOI:** 10.1192/bjo.2021.205

**Published:** 2021-06-18

**Authors:** Sarah Wordie, Alice Troup, Giovana Klefti, Cinzia Giuntoli

**Affiliations:** Royal Edinburgh Hospital

## Abstract

**Aims:**

To assess junior doctors understanding of the law surrounding the use of The Mental Health (Care and Treatment) (Scotland) Act 2003 (MHA) with a focus on assessing confidence and knowledge of the use of the emergency detention certificate (EDC). A secondary aim was to use these findings to develop a variety of educational tools to subsequently improve junior doctors understanding in using the MHA.

**Method:**

We created and distributed a comprehensive electronic survey to 152 Foundation Year Two Doctors working in NHS Lothian, Fife and Borders in December 2020. We subsequently interviewed 20 respondents to enquire about additional resources needed to improve knowledge of the MHA. Following on, we completed worked EDC exemplars, created an easily accessible guide with step-by-step instructions on implementing an EDC and devised a checklist pro-forma that can be accessed and inserted into a patient's electronic notes to ensure all necessary steps are completed for the EDC.

**Result:**

51 doctors (34%) responded to our survey, of which 10 (19%) had previously worked in psychiatry and 16 (31%) had previously completed an EDC. 27 respondents (52%) reported a lack of self-confidence and knowledge and 26 (51%) reported a lack of understanding in the legal processes as barriers faced when putting an EDC in place. 23 (45%) respondents were unaware that a Mental Health Officer (MHO) must be contacted to grant an EDC. Respondents who had experience of working in psychiatry reported greater awareness of the MHA. From the focused interviews held, colleagues requested for worked EDC examples, an easily accessible checklist with relevant contact details and an electronic pro-forma for patients notes to assist with completing the relevant legal steps when implementing an EDC.

**Conclusion:**

Our study identified a lack of confidence in understanding the MHA and completing an EDC. Our educational materials will provide an invaluable source of information for junior doctors, in particular those with little experience of the MHA. Importantly, our resources will ensure the legal aspects of implementing an EDC are both complied with and documented appropriately.

